# Omega-3 Polyunsaturated Fatty Acids and the Intestinal Epithelium—A Review

**DOI:** 10.3390/foods10010199

**Published:** 2021-01-19

**Authors:** Luke A. Durkin, Caroline E. Childs, Philip C. Calder

**Affiliations:** 1 School of Human Development and Health, Faculty of Medicine, University of Southampton, Southampton SO16 6YD, UK; c.e.childs@soton.ac.uk (C.E.C.); pcc@soton.ac.uk (P.C.C.); 2 Institute of Life Sciences, University of Southampton, Southampton SO17 1BJ, UK; 3 NIHR Southampton Biomedical Research Centre, University Hospital Southampton NHS Foundation Trust and University of Southampton, Southampton SO16 6YD, UK

**Keywords:** ω-3 PUFA, fish oil, inflammation, cytokine, chemokine, lipid mediator, eicosanoid, permeability, enterocyte, epithelium

## Abstract

Epithelial cells (enterocytes) form part of the intestinal barrier, the largest human interface between the internal and external environments, and responsible for maintaining regulated intestinal absorption and immunological control. Under inflammatory conditions, the intestinal barrier and its component enterocytes become inflamed, leading to changes in barrier histology, permeability, and chemical mediator production. Omega-3 (ω-3) polyunsaturated fatty acids (PUFAs) can influence the inflammatory state of a range of cell types, including endothelial cells, monocytes, and macrophages. This review aims to assess the current literature detailing the effects of ω-3 PUFAs on epithelial cells. Marine-derived ω-3 PUFAs, eicosapentaenoic acid and docosahexaenoic acid, as well as plant-derived alpha-linolenic acid, are incorporated into intestinal epithelial cell membranes, prevent changes to epithelial permeability, inhibit the production of pro-inflammatory cytokines and eicosanoids and induce the production of anti-inflammatory eicosanoids and docosanoids. Altered inflammatory markers have been attributed to changes in activity and/or expression of proteins involved in inflammatory signalling including nuclear factor kappa-light-chain-enhancer of activated B cells (NF-κB), peroxisome proliferator activated receptor (PPAR) α and γ, G-protein coupled receptor (GPR) 120 and cyclooxygenase (COX)-2. Effective doses for each ω-3 PUFA are difficult to determine due to inconsistencies in dose and time of exposure between different in vitro models and between in vivo and in vitro models. Further research is needed to determine the anti-inflammatory potential of less-studied ω-3 PUFAs, including docosapentaenoic acid and stearidonic acid.

## 1. Introduction

In humans, the gut (intestinal) barrier is the largest interface separating the internal and external environments. The main cells forming the barrier are epithelial cells, although other cell types are also present. Intestinal epithelial cells (enterocytes) have a vital role in uptake of nutrients and water, in protecting internal systems from noxious environmental stressors and in regulating responses to both harmful and harmless external stimuli. Epithelial cells must respond to commensal and pathogenic bacteria, co-exist and interact with intestinal immune cells, and deal with nutrients and other substances from the diet [1]. The function and integrity of the epithelial barrier are governed by the tightly regulated axis between the epithelial cells, the microbiota, mucus and anti-microbial peptides, and immune cells. Disruption of intestinal epithelium function leads to increased permeability and loss of immunological control, which is believed to contribute to a number of pathological states and diseases, including inflammatory bowel diseases (Crohn’s disease and ulcerative colitis), coeliac disease and irritable bowel syndrome [2]. Communication between epithelial cells, the gut microbiota, and intestinal immune cells is through both direct cell–cell contact and through the production of chemical mediators, including both small metabolites such as short-chain fatty acids (SCFAs) and larger immune mediators such as cytokines and chemokines [3,4].

**Figure 1:**
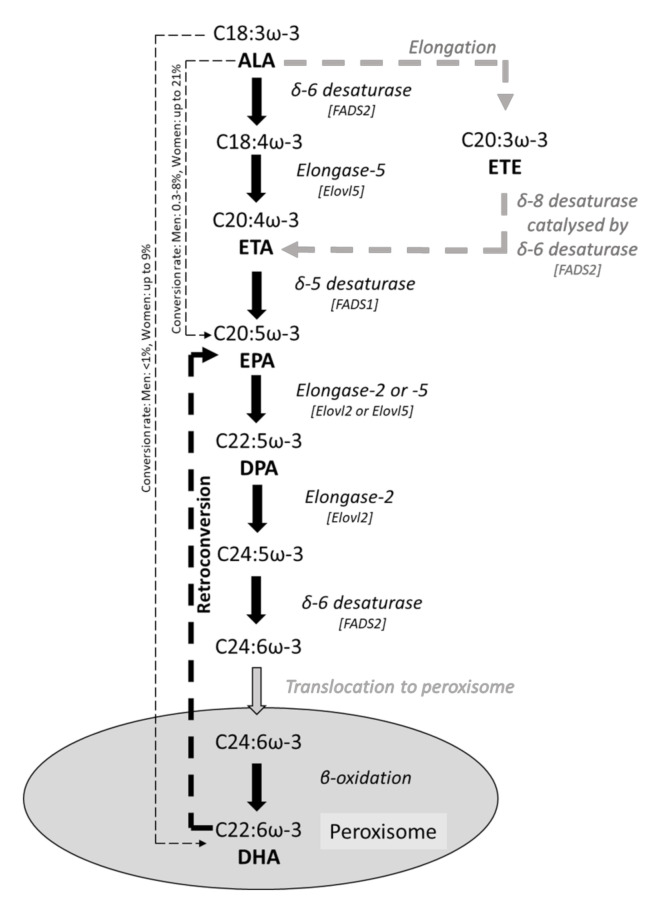
Metabolic conversion pathway from the essential ω-3 PUFA, ALA, to longer-chain ω-3 PUFAs, EPA, DPA and DHA. Conversion data are from [18].

The gut epithelium interacts with a range of nutrients and other components from the diet, which can both affect barrier function and be absorbed across the barrier into the bloodstream. The influence of diet-derived substances on gut epithelial function including barrier integrity is likely to be important. Excessive inflammation or oxidative stress can disrupt barrier function. Vitamins (including vitamins C, D_3_, and E) [5,6], phytochemicals (including polyphenols and flavonoids) [7], SCFAs [8], and polyunsaturated fatty acids (PUFAs) [9] are known to have anti-oxidant and anti-inflammatory effects on a range of cell types. Omega-3 (ω-3) PUFAs have been widely studied in the context of inflammation [9,10,11,12], with anti-inflammatory effects being described and benefits related to these being reported in several disease states, including cardiovascular disease [13], rheumatoid arthritis [14], and inflammatory bowel diseases [15]. The two main bioactive ω-3 PUFAs are eicosapentaenoic acid (EPA; 20:5ω-3) and docosahexaenoic acid (DHA; 22:6ω-3), although roles for docosapentaenoic acid (DPA; 22:5ω-3) are now emerging. These fatty acids can be consumed directly in high amounts by eating oily fish, such as salmon, mackerel and sardines, or by taking fish oil supplements. Additionally, the plant-derived ω-3 PUFA, alpha-linolenic acid (ALA; 18:3ω-3), found abundantly in flaxseeds and walnuts, and in rapeseed (sometimes called canola) and soybean oils, can be consumed as a precursor for EPA and DHA synthesis in humans, as shown in Figure 1, although humans have a relatively low conversion rate of ALA to EPA and on to DHA [16].

The mechanisms by which EPA and DHA exert anti-inflammatory effects have been described elsewhere in detail [9,10,11,12] and include partial replacement of arachidonic acid (AA; 20:4ω-6) in cellular phospholipids, inhibition of several inflammatory signalling pathways, and activation of peroxisome proliferator-activated receptor (PPAR)-γ and G-protein coupled receptor (GPR) 120, which subsequently inhibit the action of the pro-inflammatory transcription factor nuclear factor kappa-light-chain-enhancer of activated B cells (NF-κB) [17]. These anti-inflammatory effects of ω-3 PUFAs have been mostly studied in classic inflammatory cells such as monocytes, macrophages and neutrophils and also in endothelial cells and result in decreased cellular activation and reduced production of inflammatory cytokines, chemokines and lipid mediators and increased production of pro-resolving lipid mediators [9,10,11,12]. Several enzymes, known to produce mediators of inflammation such as inducible nitric oxide synthase (iNOS) and cyclooxygenase-2 (COX-2), are also known to be modulated by ω-3 PUFAs. Despite this extensive research, effects of ω-3 PUFAs on intestinal epithelial cells are less well recognised, even though controlling inflammation at the level of the intestinal epithelium is clearly important. This review aims to bring together the relevant literature assessing the role of ω-3 PUFAs, including ALA, EPA, DPA and DHA, on the function of intestinal epithelial cells with an emphasis on inflammation.

## 2. Models for Studying the Intestinal Epithelium

Directly assessing the function of the human gut epithelium in vivo is a challenge, as assessing intestinal barrier morphology, integrity and function by biopsy or endoscopy is both time consuming and invasive. Alternative indirect in vivo assessments of epithelial barrier function include permeability assays using large or small inert sugars as markers for intestinal absorption, and measuring bacterial products, such as lipopolysaccharide (LPS; also known as endotoxin), or markers of enterocyte functionality, such as citrulline and intestinal fatty acid-binding protein, within serum or plasma [19]. These less invasive approaches give insight into overall gut barrier functionality, but they cannot identify the location or cause of any differences observed, and therefore the use of complementary in vivo and in vitro techniques allows for more insight into the mechanisms of intestinal epithelium function in health and disease and for the study of the effect of stressors and modulators.

Cellular models of the intestinal epithelium provide a useful tool to investigate the potential roles of nutrients, such as ω-3 PUFAs, in vitro. Although the complexity of the intestinal barrier is difficult to replicate in vitro, multiple models are available to study intestinal epithelial cells. Human colorectal tumour-derived cell lines, such as Caco-2, T84, and HT-29, as well as rodent-derived cell lines, such as IEC-6 and IEC-18, provide replicable and high-throughput models of the intestinal epithelium [20,21], whilst organ-on-a-chip cultures have begun to replicate more in vivo-like epithelial properties and behaviours [22]. These models allow for in vitro assessment of a multitude of outcomes, including changes to membrane composition, permeability, transport processes, inflammatory mediator production, receptors and signalling pathways, the cell cycle and apoptosis.

## 3. ω-3 PUFAs and Intestinal Epithelial Fatty Acid Composition

One central mechanism linked to the regulatory properties of ω-3 PUFAs is alteration and modulation of membrane fatty acid composition [9,10,11,12]. This has been demonstrated in many cell types, including in intestinal epithelial cells (see Table 1). EPA supplementation of cultured Caco-2 cells increased their membrane content of EPA [23], and 96 h supplementation with EPA and DHA increased the respective amounts of those fatty acids in Caco-2 cells [24]. Further investigation of the incorporation of EPA, as well as of DHA and ALA, in Caco-2 and T84 cells, indicated that EPA accumulates in the phospholipid fraction, unlike DHA and ALA which accumulate in the neutral lipid pool [25]. This suggests differential metabolism and handling of ω-3 PUFAs in these cells and, further, that EPA and DHA could have different functional effects in these cells. However, in separate studies, ALA, EPA, and DHA all accumulated in T84 cell phospholipids [26], whilst EPA and DHA increased ω-3 PUFA content in T84 cell lipid rafts [27], after 48 h supplementation. It is not clear why different studies have suggested different fates of ω-PUFAs in these cells, but this might relate to methodological differences between the studies.

In addition to simply incorporating the ω-3 PUFA provided in the culture medium, bioconversion of exogenous ω-3 PUFAs can also occur in epithelial cells. This has been shown in both Caco-2 and T84 cells supplemented with 30 μM of each respective fatty acid for 7 days (summarised in Table 2). Supplementation of T84 cells with ALA significantly increased ALA and eicosatrienoic (ETE; 20:3ω-3) contents compared to untreated control cells [25]. ALA and ETE contents were also increased in ALA supplemented Caco-2 cells compared to controls [25]. The appearance of ETE suggests that elongation of ALA may be preferred in these cells rather than ALA desaturation catalysed by the FADS2 gene product which is shown in Figure 1. Additionally, EPA was significantly increased in ALA-treated Caco-2 cells, suggesting that these cells have an intact pathway of conversion of ALA to EPA. This may use the direct pathway shown in Figure 1 or, if ETE is involved, then ETE can be converted to eicosatetraenoic acid (ETA; 20:4ω-3) by delta-8 desaturation most likely catalysed by an alternative activity of the FADS2 gene product delta-6 desaturase (Figure 1). EPA supplementation significantly increased EPA content in both Caco-2 and T84 cells compared to untreated control cells [25]. EPA supplementation also significantly increased DPA content compared to controls, although the increase was much greater in Caco-2 cells compared to T84 cells [25,28]. An increase in EPA and DPA content was also associated with decreased cellular content of trans-vaccenic acid, cis-9, trans-11-conjugated linoleic acid, and oleic acid in both T84 and Caco-2 cell lines [28]. Arachidonic acid (AA) decreased in EPA-treated Caco-2 and T84 cells [25], although this was not statistically significant, whilst DHA treatment decreased AA only in the T84 cell line [25]. DHA supplementation greatly increased DHA content in both Caco-2 and T84 cells [25]. In Caco-2 cells DHA significantly increased EPA content [25], indicating the presence of a so-called retroconversion step. However, this was not seen in T84 cells where DHA supplementation decreased DPA content [25].

Changes to epithelial cell fatty acid composition after ω-3 PUFA treatment have also been reported in vivo (Table 1). Due to the complexity of obtaining intestinal biopsies, in vivo evidence from human studies on changes to fatty acid composition of epithelial cells is limited. Hawthorne et al. showed that in patients with ulcerative colitis and after 1 year of fish oil supplementation, rectal mucosa EPA content increased, but mucosal DHA content was not significantly affected [35]. However, basal levels of DHA were higher in rectal mucosa compared to EPA (1.4% vs. 0.4% total fatty acids, respectively) and approximately 4-fold more EPA was consumed compared to DHA, across the trial period. In a separate human study, 12 week fish oil supplementation in patients with inflammatory bowel disease increased colonic mucosal concentrations of EPA and DHA, which coincided with a decrease in colonic mucosal AA content [34].

Rodent models have been used to assess changes in mucosal and epithelial fatty acid compositions after dietary provision of ω-3 PUFAs (Table 1). Male Wistar rats with diets supplemented with EPA or DHA for 21 days showed increased epithelial cell phospholipid concentrations of each respective PUFA [33]. Male Sprague–Dawley rats consuming a diet containing EPA and DHA, over a similar 3 week supplementation period, also had significantly increased EPA and DHA content in small intestine tissue [31]. Additionally, rats fed a diet containing fish oil with moderate concentrations of EPA and DHA in a 2,4,6-trinitrobenzene sulphonic acid (TNBS) colitis model showed significant increases in EPA and DHA content in colonic tissue [29]. Fish oil supplementation, making up 5% of the diet of male Wistar rats for 3 weeks, was again shown to significantly increase EPA and DHA content in colonic tissue after TNBS or dextran sodium sulphate (DSS)-induced colitis treatment [32]. Rag2^−/−^ immunodeficient mice supplemented for 8 weeks with fish oil had increased free concentrations of EPA and DHA (EPA > DHA) and decreased free concentrations of AA in colonic tissue [30]. Thus, it is clear that when exposure to EPA and DHA is increased (in cell culture or in the diet of experimental animals or humans), intestinal epithelial cells incorporate those fatty acids. Often this incorporation has been reported to be at the expense of the ω-6 PUFA, AA.

## 4. ω-3 PUFAs and Intestinal Epithelial Morphology

Improvements in gut epithelial function attributed to ω-3 PUFAs are exemplified by improved histological outcomes seen in several in vivo models. Although improvements have been seen in many studies, there are also reports in which ω-3 PUFA supplementation has little or no effect on gut morphology (summarised in Table 3).

Transgenic mice capable of endogenously synthesising ω-3 PUFAs had increased concentrations of EPA, DPA, and DHA in intestinal epithelial cells, and when exposed to DSS to induce colitis, had improved histological outcomes, including increased colon length, decreased severity and thickness of inflammatory infiltrate, and decreased epithelial damage [36]. In this transgenic model, ω-3 PUFAs are generated endogenously, but many more studies have used exogenous ω-3 PUFAs as a potential preventative or therapeutic strategy in a range of inflammatory conditions of the gut.

Single-dose DHA treatment has been shown to reduce the incidence of necrotising enterocolitis in neonatal Sprague–Dawley rats, as indicated by lower numbers of villous necroses [41,43]. Moreover, prolonged DHA exposure (14 days) in the IL-10-deficiency-induced murine colitis model reduced colonic inflammation and inflammatory scores and inflammatory cell infiltration into the intestinal mucosa, and partially restored goblet and glandular architecture [46]. In a model of DSS-induced colitis, DPA also had a protective effect on disease severity and gut morphology, including attenuated colon shortening, decreased inflammatory infiltration into colonic tissue, and attenuated body weight loss, as well as overall decreases in pathology and gross morphological injury scores [52].

Fish oil, containing both EPA and DHA, has also exhibited protective effects on gut histology in other models, including reducing mucosal damage and preventing tight junction protein redistribution in a rat haemorrhagic shock model [45], as well as increasing villous length and crypt depth/villous length ratio in a female Sprague–Dawley rat model of peritoneal dialysis [51]. Fish oil also significantly increased villous height and improved histological architecture scores in β-lactoglobulin-treated female BALB/c mice, as well as preventing changes to short current circuit and tissue conductance in jejunal tissue [50]. EPA-enriched fish oil, given over a 6 week period, also reduced macroscopic inflammation parameters in an acetic acid-induced colitis model [38]. However, in chronic ethanol-exposed male Wistar rats, fish oil had no effect on histological epithelial damage [47].

In young male Sprague–Dawley rats, ω-3 PUFAs given over 28 days had no effect on TNBS colitis-induced alterations to colon length to weight ratio [49]. This is in accordance with the lack of effect seen in a TNBS-induced colitis model using male Wistar rats supplemented with fish oil [32]. Conversely, in an adult Sprague–Dawley rat model of TNBS colitis, ω-3 PUFA supplementation over 60 days decreased macroscopic parameters of inflammation, including reducing ulcerations, tissue thickening, and inflammatory cell infiltration [48]. Male Sprague–Dawley rats consuming a cod liver digest (rich in ω-3 PUFAs) over 50 days showed decreased macroscopic damage scores and absence of colonic inflammation and ulcerations in chronic TNBS-induced colitis [37]. Short-term (12 days) consumption of a ω-3 PUFA-rich diet also reduced histological inflammation initiated by TNBS [42]. Yuceyar et al. described that the mode of delivery of fish oil can influence its effectiveness in preventing histological changes in TNBS colitis. Fish oil given in the diet, over a 6 week period, was able to attenuate lesion quantity and improved pathological scores in TNBS-induced colitis, whilst the same dose given by daily enema, over a 2 week period, had no effect on macroscopic parameters of inflammation and overall pathology score [40].

In contrast to the many findings of benefit of ω-3 PUFAs described above, Reifen et al. reported that fish oil treatment increased mucosal inflammatory scores in a DSS-induced colitis model using male Wistar rats, while ALA-rich sage oil had no effect on in vivo parameters of inflammation in either TNBS- or DSS-induced colitis [32]. However, Shoda et al. showed protective effects of both fish oil and ALA-rich perilla oil in a model of TNBS-induced colitis. Both treatments altered colitis-induced histological changes, with fish oil reducing ulcer severity and perilla oil reducing the increase in colonic weight [39]. In a Sprague–Dawley rat model of TNBS colitis, ALA reduced macroscopic lesions and neutrophil infiltration, but had no effect on colitis-induced changes to wall thickness and overall inflammatory score [44]. Thus, although there is substantial literature on benefits of ω-3 PUFAs in various models of gut inflammation, there are some contrary findings which might be due to differences between models, age groups, ω-3 PUFA dose, and delivery method of ω-3 PUFAs.

## 5. ω-3 PUFAs and Intestinal Permeability

**Figure 2:**
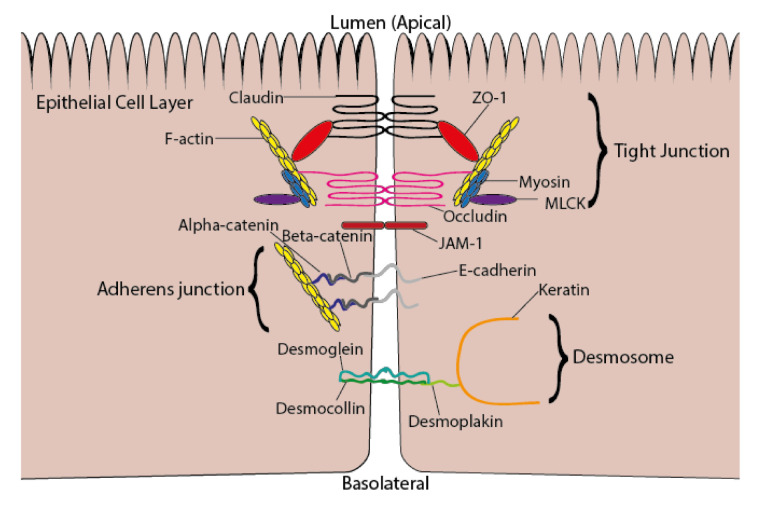
Intercellular junctions between epithelial cells consisting of tight junctions, adherens junctions, and desmosomes. Abbreviations used: ZO-1, zonula occludens-1; MLCK, myosin light chain kinase; JAM-1, junction adhesion molecule-1.

Regulated permeability is one of the vital functions of the intestinal epithelium. The structure and function of intercellular junctions, including tight junctions, are integral to maintaining the uptake of nutrients and preventing the translocation of commensal bacteria and their products across the epithelial barrier. Tight junctions consist of extracellular spanning proteins, occludin and claudin, which interact with adjacent cells at cell–cell contact sites, and anchoring protein, zonula occludens (ZO)-1, which attaches occludin and claudin to the actin cytoskeleton [53], as shown in Figure 2. Adherens junctions are intercellular adhesion structures, responsible for maintaining tissue integrity and control of epithelial cell motility and proliferation [54]. Desmosomes are adhesion sites between adjacent cells, which provide linkage to the intermediate filament cytoskeleton and provide resistance against mechanical stress [55]. Junction adhesion molecule (JAM)-1 is associated with barrier function and tight junction assembly [56]. Disruption of intercellular junctions occurs in response to increased presence of inflammatory cytokines, pathogenic bacteria, or bacterial lipopolysaccharides [53], which induce regulatory proteins, such as myosin light chain kinase (MLCK), leading to tight junction protein degradation or endocytosis [57]. Intercellular junction dysfunction can also be attributed to certain pathological conditions, including inflammatory bowel disease, obesity, and non-alcoholic fatty liver disease [53]. Nutrients from the diet, including amino acids, vitamins A and D, and polyphenols, are known to modulate intercellular junctions, in particular tight junctions [58].

The effects of ω-3 PUFAs on permeability in in vitro and in vivo models are summarised in Table 4. Usami et al. reported that ALA, EPA and DHA all reduced barrier integrity of Caco-2 cell monolayers, indicated by increased fluorescein sulfonic acid permeability and decreased transepithelial electrical resistance [59,60]. These increases in permeability could be associated to a cytotoxic level of ω-3 PUFAs being used, as high levels of EPA increased lactate dehydrogenase (LDH) release into the culture medium, a marker for cytotoxicity, in Caco-2 cell monolayers [59]. In contrast to these findings, EPA and DHA have been shown to improve barrier integrity in many other in vitro studies. In unstimulated Caco-2 cell monolayers, 24 h EPA supplementation reduced permeability of horseradish peroxidase [23]. EPA and DHA were shown to attenuate increases in permeability induced by pro-inflammatory cytokines, including interleukin (IL)-4 [26] and combined tumour necrosis factor (TNF)-α and interferon (IFN)-γ [27] in T84 cells. Additionally, EPA and DHA were shown to prevent permeability changes induced by mycotoxin, deoxynivalenol, and infection in a porcine epithelial cell model, IPEC-1, by preventing redistribution of tight junction proteins, claudin and ZO-1 [61]. Xiao et al. showed that incubation for 96 h with EPA, but not with DHA, was able to significantly attenuate increased permeability in heat stress-impaired Caco-2 monolayers [24].

Differing effects of EPA and DHA on gut permeability have been reported in vivo. Male Wistar rats subjected to heatstroke showed increased gut permeability, indicated by increased plasma D-lactate and endotoxin levels, which was attenuated by DHA, and even more effectively by EPA [33]. High-dose fish oil supplementation, containing both EPA and DHA and making up 15% total calories, prevented increased plasma endotoxin levels and decreased tight junction protein (ZO-1) expression in chronic ethanol-exposed male Wistar rats [47]. DHA also prevented increased plasma endotoxin levels in a necrotising enterocolitis model using Sprague–Dawley rats [41]. Dietary ω-3 PUFA supplementation in young male Sprague–Dawley rats had no effect on colitis-induced reductions in tight junction protein expression (claudin-1 and occludin) and barrier-associated protein expression (trefoil factor 3 and mucin 2) [49]. In other models of colitis, consumption of EPA-enriched fish oil protected intestinal absorption function [38] and transgenic mice with increased mucosal ω-3 PUFAs maintained ZO-1 tight junction protein expression [36]. Therefore, it seems the effects on ω-3 PUFAs on epithelial cell permeability may be dependent on the type of stimulus exerting changes to permeability, but could also be attributed to the dose and type of ω-3 PUFA used.

## 6. ω-3 PUFAs and Intestinal Epithelial Inflammation

Epithelial cells respond to a plethora of stimuli, both from luminal contents and the inflammatory milieu of the intestinal mucosa. The production of inflammatory mediators is governed by the cellular response to particular stimuli, and can be modulated by ω-3 PUFAs. The effects of ω-3 PUFAs on inflammatory mediator production by intestinal epithelial cells are summarised in Table 5.

Wang et al. demonstrated that, under non-inflammatory conditions, long-term (up to 90 days) fish oil consumption (40 g/kg body weight, containing 15.4% EPA and 15.1% DHA) in male Lewis rats significantly reduced cytokine mRNAs in the intestinal epithelium, including TNF-α, IFN-γ, IL-4, IL-10, and IL-15, as well as IL-15 protein expression, but had no effect on IL-7, and altered the phenotype of intraepithelial lymphocytes [62]. Acute exposure (12 h) to EPA or to DHA significantly upregulated transforming growth factor (TGF)-β1 mRNA expression in HT29 cells, and EPA, but not DHA, treatment induced a significant increase in TGF-β1 mRNA expression in mucus-secreting HT29-MTX cells [63]. Neither EPA nor DHA had a consistent effect on IL-8 and HSP 72 mRNA expression in HT29 or HT29-MTX cell cultures [63].

**Figure 3:**
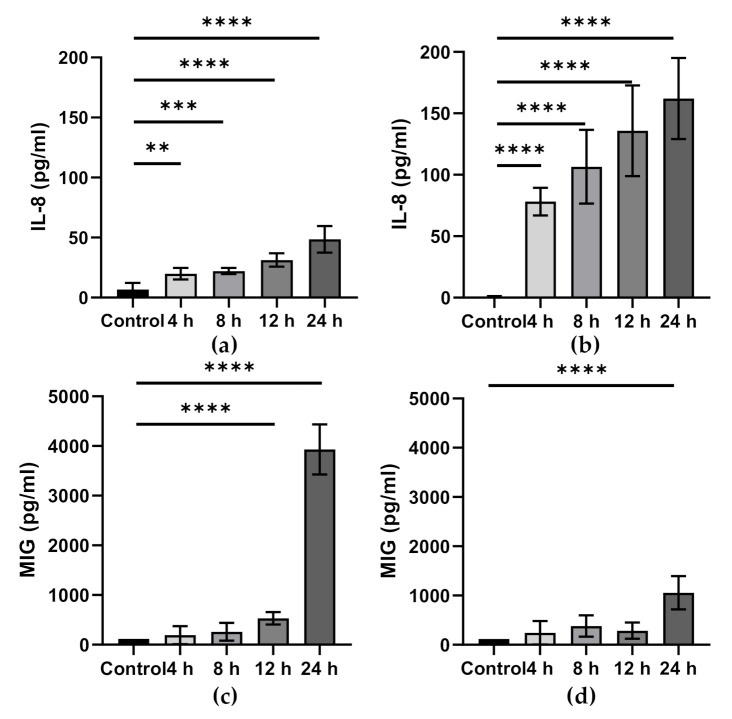
IL-8 and monokine induced by gamma interferon (MIG) production by Caco-2 cells stimulated with a cocktail of cytokines (TNF-α (5 ng/mL), IFN-γ (50 ng/mL), and IL-1β) in a Transwell system; cytokines were added to the basolateral compartment, and apical and basolateral supernatants assessed for IL-8 and MIG by Luminex. Controls are unstimulated. (**a**): Apical IL-8 production. (**b**): Basolateral IL-8 production. (**c**): Apical MIG production. (**d**): Basolateral MIG production. Data are the mean ± SEM from three experiments and are not previously published. Significance was determined by one-way ANOVA with Dunnett’s multiple comparison tests; ** *p* < 0.01, *** *p* < 0.001, and **** *p* < 0.0001.

Inflammatory challenge alters epithelial cell inflammatory mediator production, as shown in Figure 3. This is considered to be damaging to epithelial barrier integrity, and it is thought that ω-3 PUFAs can regulate these processes. Pre-incubation with EPA and DHA, but not ALA, significantly prevented IL-1β-induced increases in IL-6 and IL-8 production in Caco-2 cells [64]. In a separate model using necrotising enterocolitis epithelial cells (H4 and NEC-IEC) and Caco-2 cells, IL-8 mRNA and protein expression were attenuated by DHA, after IL-1β treatment, in all cell lines [65]. IL-1β-induced increases in IL-6 mRNA and protein were attenuated by DHA and EPA but only in H4 cells [65]. In a model of IL-1β-induced inflammation in Caco-2 cells, both ALA-rich sage oil and ALA inhibited increased IL-8 expression [32]. Separate DHA or EPA supplementation inhibited increased IL-8 expression in a colonic epithelial cell line, HCT116, treated with the nucleotide-binding oligomerization domain-containing protein (NOD) 2 ligand, muramyldipeptide (MDP) [66]. DHA, but not EPA, supplementation also inhibited increased HCT116 IL-8 expression induced by lauric acid and the nucleotide-binding oligomerization domain-containing protein (NOD) 1 agonist, γ-D-glutamyl-mesodiaminopimelic acid (IE-DAP) [66]. DHA also reduced prostaglandin (PG) E_2_ and IL-8 production in Caco-2 cells stimulated with a digest of α-gliadin [67].

In an in vivo TNBS colitis model, ALA-rich camelina oil supplementation had no effect on IL-1β expression and secretion and the production of PGE_2_, but did significantly reduce TNF-α mRNA and protein, as well as leukotriene (LT) B_4_ production, in colonic tissue [44]. IL-10-deficient mice supplemented with DHA for 14 days had significantly reduced expression of TNF-α, IFN-γ, and IL-17 in the colonic mucosa [46]. Male Sprague–Dawley rats fed EPA and DHA for 3 weeks, before intestinal reperfusion and ischaemia, showed no alteration in cytokine production [31]. However, EPA-derived metabolites including thromboxane (TX) B_3_, 17,18-epoxyeicosatetraenoic acid (EEP), and 8-iso PGF_3α_ were significantly increased in intestinal tissue [31].

Hillier et al. demonstrated that humans given fish oil over a 12 week period had attenuated colonic levels of AA-derived metabolites, PGE_2_ and TXB_2_ [34]. However, ω-3 PUFA supplementation in male Sprague–Dawley rats showed a large (30-fold) increase in PGE_2_ and a smaller increase in LTB_4_ [38]. This is unexpected, as PGE_2_ synthesis is attributed to COX-2 processing of AA, and ω-3 PUFAs reduce AA availability and inhibit its metabolism by COX-2. EPA can also be processed by COX-2 to PGE_3_, which is known to be cross-reactive in PGE_2_ assays and may explain the large increase in “PGE_2_” seen. Alternatively, after ω-3 PUFA supplementation, free AA released from cell membranes as a result of ω-3 PUFA incorporation could be processed by COX-2 to form PGE_2_, contributing to the increase in PGE_2_ seen.

In TNBS colitis models, rats fed a diet rich in ω-3 PUFAs had decreased colonic concentrations of IL-2 and IL-4 [48], as well as IL-6, but there was no effect on TNF-α [42,49]. In a mouse model of immunodeficiency-induced colitis, 8 week fish oil supplementation had no effect on mucosal PGE_2_, TXB_2_ or LTB_4_, but upregulated other colonic inflammatory mediators, including TNF-α, IL-1β, IL-12, myeloperoxidase (MPO), and keratinocyte-derived chemokine [30]. EPA- and DHA-derived metabolites including PGE_3_, TXB_3_, LTB_5_, 5-hydroxyeicosapentaenoic acid (HEPE), and 17,18-EEP were increased after fish oil supplementation, whilst AA-derived metabolites PGJ_2_, 5,6-epoxyeicosatrienoic acid (EET), 8,9-EET, and 14,15-EET were decreased [30]. In a separate colitis model, fish oil given through the diet or by enema also attenuated colonic levels of AA-derived metabolites, LTB_4_ and LTC_4_ [40].

DPA also altered inflammatory mediator production in a model of colitis in C57 mice. DPA supplementation for 4 weeks decreased colonic IL-1β, IL-6, and TNF-α mRNA and protein, as well as increasing colonic IL-10 [52]. Increases in AA-derived metabolites, PGE_2_ and LTB_4,_ were also attenuated by DPA supplementation, suggesting that DPA, like EPA and DHA, competitively inhibits AA metabolism by COX and lipoxygenase (LOX) enzymes [52]. In a model of DSS colitis, mice with elevated epithelial cell levels of EPA, DPA and DHA as a result of a transgenic manipulation, also had increased mucosal levels of ω-3 PUFA-derived metabolites, including resolvin D3, resolvin E1, protectin D1, PGE_3_, and LTB_5_ [36]. However, AA-derived metabolites, LTB_4_, PGE_2_, and 15-hydroxyeicosatetranoic acid, were unaffected. The increase in TNF-α and IL-1β mRNA in response to DSS was attenuated in these mice, whilst toll-interacting protein and trefoil factor 3 mRNAs were upregulated in colonic mucosa [36].

Overall, supplementation with ω-3 PUFAs seems to upregulate the production of pro-resolving ω-3-derived mediators through COX and LOX enzyme processing and modulation of inflammatory pathways, as well as attenuating the production of pro-inflammatory chemokines, cytokines and eicosanoids. Varying effects seen between ω-3 PUFA treatments could be associated to the mechanisms of the specific inflammatory condition, the dose of ω-3 PUFAs used, and the time of exposure to the ω-3 PUFA supplementation.

## 7. Effect of ω-3 PUFAs on Inflammatory Signalling Pathways in Intestinal Epithelial Cells

The attenuation of inflammatory mediator production (as described above) is associated with the incorporation of ω-3 PUFAs into membranes, but ω-3 PUFAs also interact with surface membrane and intracellular receptors and intrinsic inflammatory pathway regulators; these effects are summarised Table 6.

DHA induced significant increases in PPAR-α and PPAR-γ activity, which coincided with significantly decreased triglyceride and apolipoprotein B release by Caco-2 cells [68]. EPA also induced significant upregulation of PPAR-α, but had no significant effect on PPAR-γ. EPA and DHA had no effect on PPAR-δ activity in vitro [68]. Additionally, post-prandial triglyceride release in vivo was also inhibited by DHA treatment (but not by EPA) through a PPAR-α-dependent mechanism, as this effect was diminished in PPAR-α-deficient C57BL/6 mice [68]. The relevance of these findings is that PPARs, especially PPAR-γ, are anti-inflammatory and these studies show that they can be activated by EPA and DHA. In a separate model, PPAR-γ expression was upregulated by both EPA and DHA in IL-1β-treated Caco-2 cells [64]. ALA had no effect on PPAR-γ expression in either IL-1β-stimulated Caco-2 cells [64] or colonic tissue of TNBS-treated male Sprague–Dawley rats [44]. Additionally, EPA had no effect on PPAR-α mRNA expression in either Caco-2 or T84 epithelial cell lines [28]. Although variable effects have been seen on PPAR expression, assessing the activation of these receptors, through associated downstream mechanisms, such as NF-κB and iNOS, gives further insight into the action of ω-3 PUFAs.

DHA attenuated NF-κB activation and the degradation of the NF-κB inhibitory subunit, IκBα, induced by lauric acid, MDP, and IE-DAP treatment in HCT116 cells, whilst EPA had no effect [66]. DHA, but not EPA, attenuated NF-κB and IL-1R1 mRNA expression in IL-1β-treated H4 and Caco-2 cells [65]. ALA supplementation inhibited the activation of NF-κB and expression of the oxidative stress mediator, iNOS, in colonic tissue of TNBS-treated male Sprague–Dawley rats but had no effect on phosphorylation of downstream regulators, including JNK, P38, and IκB [44]. Increased ω-3 PUFA content of colonic tissue, through transgenic modification, attenuated increased NF-κB activation and iNOS mRNA expression [36]. IL-1β treatment induced iNOS expression in Caco-2 cells, which was attenuated by EPA and DHA supplementation, but not by ALA supplementation [64]. Conversely, ALA supplementation in a similar model, using IL-1β-stimulated Caco-2 cells, significantly reduced iNOS expression [32] and DHA supplementation had no effect on iNOS expression in colonic tissue from a neonatal Sprague–Dawley rat necrotising enterocolitis model [41]. Disparate findings from these studies could be linked to ω-3 PUFA dose or to the precise inflammatory mechanisms involved in the different models, as necrotising enterocolitis is driven by a multitude of inflammatory mediators, including platelet-activating factor (PAF), TNF-α, and a variety of interleukins [69].

COX-2 activity and expression, which is involved in the production of PGs from AA, can be regulated by ω-3 PUFAs. In male Wistar rats treated with TNBS or DSS to induce colitis, both fish oil and sage oil (rich in ALA) decreased COX-2 mRNA expression in colonic tissue [32]. ALA supplementation in TNBS-stimulated Sprague–Dawley rats also decreased COX-2 expression in colonic tissue [44]. Additionally in vitro, ALA and sage oil treatment inhibited COX-2 expression in IL-1β-stimulated Caco-2 cells [32] and DHA supplementation of Caco-2 cells stimulated with α-gliadin also reduced COX-2 expression [67]. ω-3 PUFA supplementation suppressed colonic COX-2 expression, as well as increasing IL-1A, toll-like receptor (TLR) 2, and mitogen-activated protein kinase kinase 3 mRNA expression in a Sprague–Dawley rat model of TNBS-induced colitis [49]. DHA has also been shown to inhibit toll-like receptor 4 and PAF receptor mRNA expression in PAF-treated IEC-6 epithelial cultures [43]. PAF receptor and phospholipase A_2_ expression were also suppressed by DHA in a neonatal Sprague–Dawley rat model of necrotising enterocolitis [41]. DHA treatment in HT-29 epithelial cultures altered cell maturation, with fewer cells in the stationary phase and increased numbers of cells in the G_0_/G_1_ phase of the cell cycle. Additionally, DHA potentiated TNF-α and anti-Fas antibody induced epithelial cell apoptosis [70]. Dietary fish oil supplementation in male Wistar rats reduced MPO activity in a model of TNBS colitis, whilst the same dose of fish oil given by enema had no effect on MPO activity [40]. It seems that the route or the mode of administration may affect the anti-inflammatory efficacy of fish oil in this model of colitis.

Microarray analysis has also shown that EPA and DHA can regulate a multitude of pathways across small intestinal tissue. Six hours after being given an oral dose of either EPA or DHA, small intestinal tissue resected from wild-type mice showed regulated responses to pathways involved in long-chain fatty acid uptake, peroxisomal β-oxidation, ω-oxidation, metabolism of energy-yielding substrates, and oxidative stress, as well as suppression of the cholesterol uptake transporter, Npc1l1, the apical mannose and glucose uptake transporter, Sglt4, and the serotonin transporter, Slc6a4. EPA treatment (but not DHA) also increased cholesterol efflux protein, Abca1, and dopamine transporter, Dat1, expression [71].

## 8. Gut Microbiota, Gut Inflammation, and ω-3 PUFAs

In addition to direct effects of ω-3 PUFAs on intestinal epithelial inflammation, it is important to consider the contribution of the microbiota to the regulation and maintenance of the gut epithelium–immune system axis and whether ω-3 PUFAs might mediate some of their actions via effects on the microbiota. Homeostatic mechanisms of the gut microbiota include anti-microbial protection, immunomodulation (through secretion of mediators and interaction with epithelial cells), maintenance of intestinal barrier integrity, and nutrient metabolism [72]. Microbial dysbiosis can contribute to the dysfunction of the intestinal barrier and the induction of intestinal inflammation [73]. Supplementation with ω-3 PUFAs has been shown to alter the composition of the gut microbiota across the life course in mouse models [74,75]; however, the evidence for ω-3 PUFA-induced modulation of the gut microbiota in humans is still relatively scarce. Djuric et al. have recently described that ω-3 PUFA consumption induced small changes to gut microbial populations in humans, which were associated with increased colonic EPA to arachidonic acid ratios and a reduction in colonic PGE_2_ expression [76]. Associations between increased favourable gut microbial populations and improved disease outcomes with ω-3 PUFA treatment have also been reported in obesity [77], rheumatoid arthritis [78] and cancer [79]. The microbial populations that appear to be promoted by increased ω-3 PUFA intake, including *Bacteroidetes*, *Firmicutes*, *Lachnospiraceae*, *Bifidobacteria*, and *Enterobacteria*, exhibit anti-inflammatory properties through increased production of SCFAs, particularly butyrate, and reduced endotoxaemia [80]. There is evidence that ω-3 PUFAs can be metabolised by particular bacterial species, resulting in the production of potential active metabolites, which could induce anti-inflammatory actions in the intestinal mucosa [81,82]. Further research into EPA, DPA and DHA metabolism by the gut microbiota and the subsequent effects on the gut epithelium could identify new anti-inflammatory mechanisms for ω-3 PUFAs.

## 9. ω-3 PUFAs and Human IBD

The pre-clinical research involving cell lines and rodent models described in earlier sections demonstrates the ability of ω-3 PUFAs to exert anti-inflammatory actions at the level of the intestinal epithelium. This would suggest a role for ω-3 PUFAs in preventing, and perhaps in treating, human inflammatory bowel diseases (IBD). Mozaffari et al. recently conducted a systematic review and meta-analysis of studies evaluating intake of fish or of different ω-3 PUFAs and risk of incident IBD [83]; studies were a mix of prospective cohort studies and case–control studies. An inverse association between fish consumption and the incidence of Crohn’s disease (effect size: 0.54) was observed, but there was no relationship between total dietary ω-3 PUFA intake and risk of IBD (effect size: 1.17) [83]. There was a significant inverse association between dietary EPA and DHA intake and the risk of IBD (effect size: 0.78) and ulcerative colitis (effect size: 0.75), but not of Crohn’s disease (effect size: 0.85). There was no association between ALA intake and IBD (effect size: 1.17) [83]. Thus, EPA and DHA, but not ALA, may protect against development of ulcerative colitis. With regard to the treatment of IBD, there have been a number of trials of ω-3 PUFAs (mainly preparations of EPA plus DHA) over the years; trials conducted prior to 2009 are reviewed in [84] while trials published since 2010 are reviewed in [85]. The focus of the majority of such human trials has not been on the function of the intestinal barrier and epithelial cells specifically, but rather on macroscopic inflammatory scores, relapse and remission rates, and systemic markers of inflammation such as plasma concentrations of cytokines. The findings from these trials are inconsistent. Several systematic reviews and meta-analyses of randomised controlled trials of ω-3 PUFAs in patients with IBD have been published [86,87,88,89]. Ajabnoor et al. recently published a large meta-analysis of 83 randomised controlled trials reporting on inflammatory outcomes and including over 41,000 participants [89]. Of these trials, 13 recruited patients with IBD. The meta-analysis identified that increasing intake of EPA and DHA may reduce risk of IBD relapse (relative risk 0.85) and of IBD worsening (relative risk 0.85); EPA and DHA also reduced erythrocyte sedimentation rate, a marker of inflammation in patients with IBD [89]. ALA seemed to have little effect. EPA and DHA supplementation was also seen to increase risk of IBD diagnosis and to increase faecal calprotectin (a biomarker of IBD) [89], effects which are difficult to explain and which conflict with the findings of the meta-analysis of Mozaffari et al. [83].

## 10. Summary, Concluding Remarks and Limitations of the Literature

**Figure 4:**
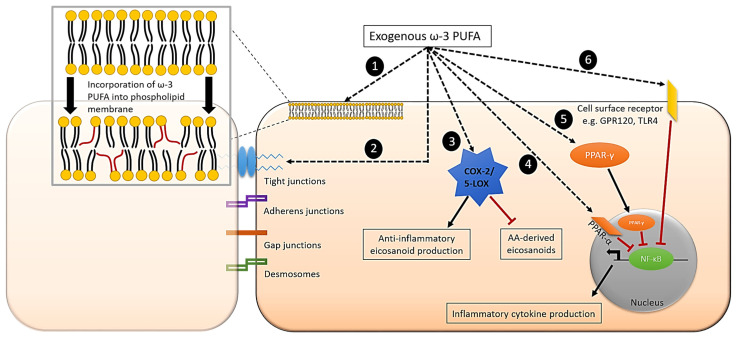
Proposed mechanisms involved in ω-3 PUFA regulation of inflammation in intestinal epithelial cells. (**1**). Incorporation of ω-3 PUFA into phospholipid membrane/lipid rafts. (**2**). Modulation of tight junction protein expression and redistribution. (**3**). Production of anti-inflammatory eicosanoids and inhibition of AA-derived eicosanoids catalysed by COX-2 or 5-LOX. (**4**). Activation of nuclear receptors, e.g., PPAR-α. (**5**). Translocation of transcription factors into nucleus, e.g., PPAR-γ. (**6**). Interaction with transmembrane/cell surface receptors, e.g., G-protein coupled receptor 120 (GPR120) and TLR4. Mechanisms 4, 5, and 6 lead to the inhibition of NF-κB and the subsequent reduced production of multiple inflammatory mediators.

Epithelial cells appear to easily incorporate ω-3 PUFAs both in vitro and in vivo. They also metabolise ALA to EPA and DHA. Overall, the literature supports the regulatory properties of marine-derived ω-3 PUFAs, EPA, DPA and DHA, and the plant-derived ω-3 PUFA, ALA, in intestinal epithelial cells and tissue. This regulatory role of ω-3 PUFAs in epithelial cells involves a number of mechanisms (Figure 4), and these fatty acids appear to reduce responses of epithelial cells to many inflammatory stimuli both in vitro and in vivo and lead to improvements in inflammation-related outcomes in many animal models as well as in patients. EPA and DHA are incorporated fairly rapidly into cultured epithelial cells (over hours to days). Rodent models showed increased intestinal tissue ω-3 PUFA composition after fish oil consumption, over longer supplementation periods (7–21 days), but shorter periods have not been frequently investigated. Two human studies indicated that fish oil supplementation over 12 weeks and 1 year altered ω-3 PUFA content in colonic and rectal tissue, respectively [34,35]. Therefore, it seems that complexity of the model (human > rodent > cell) is correlated with the time period required to alter ω-3 PUFA content of intestinal tissue.

Histological improvements after ω-3 PUFA supplementation in rodents have been reported in a range of inflammatory conditions, including various forms of colitis, haemorrhagic shock, IL-10 deficiency, β-lactoglobulin-induced inflammation, and peritoneal colitis. However, in some studies of colitis and a model of chronic ethanol exposure, no benefit of ω-3 PUFAs was seen. The specific protective effect or lack of effect seen could be attributed to the specific ω-3 PUFA used, the dose of ω-3 PUFAs, the inflammatory condition, or a combination of these factors.

Investigations using cell and rodent models have explored the effects of ω-3 PUFAs on specific inflammatory outcomes including permeability, inflammatory mediator production, and regulation of inflammatory proteins. ω-3 PUFA supplementation was shown to influence permeability by preventing tight junction protein redistribution or changes to tight junction protein expression in cell models of cytokine-induced inflammation, heat stress, and deoxynivalenol-induced inflammation, and in rodent models of DSS-induced and acetic acid-induced colitis, necrotising enterocolitis, heatstroke, and chronic ethanol exposure. However, in one rodent model of TNBS-induced colitis, ω-3 PUFA treatment had no effect on permeability-associated protein expression [49].

Inflammatory cytokines, such as IL-6 and IL-8, were attenuated by ω-3 PUFA supplementation in several different inflammatory conditions, including IL-1β-induced inflammation, lauric acid-induced inflammation, α-gliadin-induced inflammation, and TNBS-induced colitis. Additionally, PUFA-derived metabolites were altered after ω-3 PUFA supplementation, although the findings varied across different models. Inflammatory regulators responsible for alterations in mediator production, such as NF-κB, iNOS, COX-2, and PPARs, were altered by ω-3 PUFA supplementation in various cell and rodent models.

Thus, the current body of literature highlights a large range of interacting mechanisms of action of ω-3 PUFAs by which they reduce epithelial inflammation; these mechanisms are identified in both cell models and in rodents. Nevertheless, it remains unclear which ω-3 PUFA is most effective, although comparator studies suggest this might be DHA, and the actual primary mechanism(s) within each different inflammatory condition is not clear. Further to this, ω-3 PUFAs modify the gut microbiota in ways that might reduce epithelial inflammation. In addition, gut microbes can metabolise ω-3 PUFAs to bioactive anti-inflammatory mediators. Through their multiple anti-inflammatory actions, ω-3 PUFAs, especially EPA and DHA, would be expected to reduce risk of human IBD and perhaps even improve outcomes in patients with existing IBD. In both regards, the existing literature is inconsistent. However, recent systematic reviews and meta-analyses indicate that EPA and DHA, but not ALA, may protect against development of ulcerative colitis [83] and may reduce the risk of IBD relapse and worsening [89].

One limitation of the current literature is the large variety of concentrations/doses of ω-3 PUFAs used across the different models. In some cases, the concentrations used in in vitro models and the doses used in in vivo rodent models are extremely high and therefore hard to translate to the human equivalent diet. A second limitation is a lack of human studies investigating the intestinal epithelium in the context of altered ω-3 PUFA exposure. Most studies have focused on EPA and DHA, with a number of in vitro and some animal studies of ALA. DPA is emerging as a bioactive ω-3 PUFA. Epithelial cells produce DPA [25,28] from EPA and there have been a small number of studies of DPA in intestinal epithelial models [36,52]; however, DPA requires greater investigation in this context. Other underexplored ω-3 PUFAs such as stearidonic acid (18:4 ω-3) also deserve investigation using these models.

## Figures and Tables

**Table 1:** Effects of ω-3 PUFAs on fatty acid composition in intestinal cell models or tissue.

Reference	Model Used	Condition	ω-3 PUFA(s) Used	Concentration or Dose Used	Duration	Change in Fatty Acid Composition
Cell line models						
Rosella et al. [23]	Caco-2 cells	Non-stimulated	EPA	100 μg/mL	24 h	↑ Membrane EPA content
Renaville et al. [28]	T84/Caco-2 cells	Non-stimulated	EPA	30 or 300 μM	3 h or 3 h and 7 days	↑ Cellular EPA content ↑ Cellular DPA content ↓ Cellular *trans*-vaccenic acid content ↓ Cellular *cis*-9, *trans*-11-conjugated linoleic acid ↓ Cellular oleic acid content
Willemsen et al. [26]	T84 cells	IL-4-induced inflammation	ALA	10 or 100 μM	96 h	↑ Phospholipid ALA content
			EPA			↑ Phospholipid EPA content
			DHA			↑ Phospholipid DHA content
Li et al. [27]	T84 cells	TNF-α- and IFN-γ-induced inflammation	EPA	25–75 μM	48 h	↑ Lipid raft EPA content
			DHA			↑ Lipid raft DHA content
Xiao et al. [24]	Caco-2 cells	Heat stress	EPA	50 μM	96 h	↑ Membrane EPA content
			DHA			↑ Membrane DHA content
Beguin et al. [25]	T84 cells	Non-stimulated	ALA	30 μM	7 days	↑ Cellular ALA content ↑ Cellular ETE content
			EPA			↑ Cellular EPA content ↑ Cellular DPA content
			DHA			↑ Cellular DHA content ↓ Cellular DPA content
	Caco-2 cells	Non-stimulated	ALA			↑ Cellular ALA content ↑ Cellular ETE content ↑ Cellular EPA content
			EPA			↑ Cellular EPA content ↑ Cellular DPA content
			DHA			↑ Cellular DHA content ↑ Cellular EPA content
Rodent models						
Nieto et al. [29]	Rats	TNBS colitis	Fish oil	EPA: 4.16% of dietary fatty acids DHA: 3.01% of dietary fatty acids	7 or 14 days	↑ Colonic tissue EPA content ↑ Colonic tissue DHA content
Bosco et al. [30]	Rag2^−/−^ immunodeficient mice	Adoptive transfer of naïve T-cell-induced colitis	Fish oil	EPA: 3.37 g/100 g diet DHA: 2.10 g/100 g diet	8 weeks	↑ Colonic free EPA (7.2-fold) ↑ Colonic free DHA (2.2-fold) ↓ Colonic free arachidonic acid
Brahmbhatt et al. [31]	Male Sprague–Dawley rats	Intestinal reperfusion and ischaemia	Fish oil	EPA: 3.00% of dietary fatty acids DHA: 1.98% of dietary fatty acids	21 days	↑ Small intestine tissue EPA content ↑ Small intestine tissue DHA content
Reifen et al. [32]	Male Wistar rats	TNBS- or DSS-induced colitis	Fish oil	Fish oil: 5% by weight of total diet (EPA: 11.7% of total fatty acids DHA: 15.7% of total fatty acids)	21 days	↑ Colonic tissue EPA content ↑ Colonic tissue DHA content
Xiao et al. [33]	Male Wistar rats	Heatstroke	EPA	1 g/kg body weight per day by gavage	21 days	↑ Ileal phospholipid EPA content
			DHA			↑ Ileal phospholipid DHA content
Human studies						
Hillier et al. [34]	Human	Inflammatory bowel disease	Fish oil	Fish oil: 18 g/day (3.3 g EPA + 2.2 g DHA/day)	12 weeks	↑ Colonic mucosa EPA content ↑ Colonic mucosa DHA content ↓ Colonic mucosa AA content
Hawthorne et al. [35]	Human	Inflammatory bowel disease	Fish oil	Fish oil: 20 mL/day (4 g EPA + 1.2 g DHA/day)	1 year	↑ Rectal mucosa EPA content

Abbreviations used: ALA, α-linolenic acid; DHA, docosahexaenoic acid; DPA, docosapentaenoic acid; DSS, dextran sodium sulphate; EPA, eicosapentaenoic acid; ETE, eicosatrienoic acid; IFN, interferon; IL, interleukin; PUFA, polyunsaturated fatty acid; TNBS, 2,4,6-trinitrobenzenesulfonic acid; TNF, tumour necrosis factor.

**Table 2:** Fatty acid content changes in gut epithelial cell lines (T84 and Caco-2) after 7 days of supplementation with ALA, EPA or DHA. Fatty acid content is given as mean picomoles of fatty acid per microgram of protein ± standard error, () indicate fold change compared to control. * Significant differences between treatment and control. Data are from [25].

	Control (No Added ω-3 PUFA)		ALA Treatment		EPA Treatment		DHA Treatment	
Cell line	T84	Caco-2	T84	Caco-2	T84	Caco-2	T84	Caco-2
ALA	4.5 ± 1.2	30.1 ± 6.9	144.5 ± 0.6 * (32.1)	414.9 ± 40.1 * (13.8)	1.4 ± 0.6 (−0.3)	31.5 ± 4.7 (1.0)	2.3 ± 1.1 (−0.5)	41.3 ± 4.2 (1.4)
ETE	0.7 ± 0.4	7.0 ± 0.8	3.9 ± 0.3 * (5.6)	83.1 ± 9.3 * (11.9)	0.3 ± 0.3 (−0.4)	7.5 ± 0.8 (1.1)	Not detected	7.5 ± 0.8 (1.1)
EPA	9.5 ± 1.6	15.4 ± 1.9	8.9 ± 10.2 (−0.9)	20.7 ± 2.3 * (1.3)	130.8 ± 11.1 * (13.8)	257.6 ± 37.2 * (16.7)	17.0 ± 5.9 (1.8)	32.3 ± 4.4 * (2.1)
n-3 DPA	5.7 ± 1.2	19.9 ± 2.0	4.5 ± 1.3 (−0.8)	21.6 ± 2.1 (1.1)	10.6 ± 1.4 * (1.9)	157.4 ± 23.2 * (7.9)	1.1 ± 0.5 * (−0.2)	24.9 ± 3.3 (1.3)
DHA	11.8 ± 1.8	37.2 ± 4.1	9.8 ± 0.7 (−0.8)	40.8 ± 3.9 (1.1)	7.4 ± 0.7 (−0.6)	33.9 ± 4.9 (−0.9)	114.5 * ± 32.4 (9.7)	478.3 ± 58.7 * (12.9)
Total ω-3 PUFAs	32.3 ± 6.3	72.5 ± 8.1	171.6 ± 13.0 * (5.3)	581.2 ± 8.3 * (8.0)	150.7 ± 14.2 * (4.7)	448.9 ± 65.3 * (6.2)	135.0 ± 39.9 * (4.2)	535.5 ± 66.4 * (7.4)

Abbreviations used: ALA, α-linolenic acid; DHA, docosahexaenoic acid; DPA, docosapentaenoic acid; EPA, eicosapentaenoic acid; ETE, eicosatrienoic acid.

**Table 3:** Effects of ω-3 PUFAs on intestinal histology in rodent models.

Reference	Model Used	Condition	ω-3 PUFA(s) Used	Dose Used	Duration	Histological Changes
Vilaseca et al. [37]	Male Sprague–Dawley rats	Chronic TNBS colitis	Cod liver digest (providing EPA and DHA)	EPA: 5.95 mg/g diet DHA 6.91 mg/g diet	50 days	Decreased macroscopic damage (after day 20) Absence of inflammation and ulcerations (day 50)
Empey et al. [38]	Male Sprague–Dawley rats	Acetic acid-induced colitis	EPA-enriched fish oil	EPA-enriched fish oil: 10% by weight of total diet	6 weeks	Improved histology and less macroscopic injury
Shoda et al. [39]	Rats	TNBS colitis	Fish oil	Fish oil: 2% by weight of total diet	Not given	Reduced ulcer severity (correlated with decreased plasma LTB_4_)
			ALA-rich perilla oil	Perilla oil: 2% by weight of total diet		Decreased colonic weight (correlated with decreased plasma LTB_4_; ALA > fish oil)
Yuceyar et al. [40]	Male Wistar albino rats	TNBS colitis	Fish oil	EPA: 14.4 mg/g diet DHA: 11.6 mg/g diet	6 weeks (diet)	Improved pathology (decreased number of lesions)
					14 days (daily enema)	No effect on macroscopic parameters No effect on pathology
Caplan et al. [41]	Neonatal Sprague–Dawley rats	Necrotising enterocolitis	DHA	23 mg/100 mL formula	96 h	Improved histological necrotising enterocolitis outcomes
Andoh et al. [42]	Male Sprague–Dawley rats	TNBS colitis	ω-3 PUFA-rich liquid diet (providing ALA)	150 mg/100 kcal	12 days (followed by 2 days starvation)	Reduced inflammatory damage score
Hudert et al. [36]	Transgenic fat-1 mice	DSS colitis	-	Mice have higher colonic EPA, DPA and DHA than controls	-	Increased colon length Decreased severity and thickness of inflammatory infiltrate Decreased epithelial damage
Lu et al. [43]	Neonatal Sprague–Dawley rats	Necrotising enterocolitis	DHA	0.5% of total fatty acids in formula	72 h	Improved histology
Hassan et al. [44]	Male Sprague–Dawley rats	TNBS colitis	ALA	28.8% of total fatty acids in formula	14 days	Decreased macroscopic lesions Less neutrophil infiltration No effect on mucosal wall thickness No effect on overall inflammatory score
Bosco et al. [30]	Rag2^−/−^ immunodeficient mice	Adoptive transfer of naïve T-cell-induced colitis	Fish oil	EPA: 3.37 g/100 g diet DHA: 2.10 g/100 g diet	8 weeks	No effect on macroscopic parameters of colitis
Li et al. [45]	Male rats	Haemorrhagic shock	Fish oil	Fish oil: 0.2 g/kg body weight	Single intravenous treatment	Less mucosal damage Improved tight junction morphology
Reifen et al. [32]	Male Wistar rats	TNBS- or DSS-induced colitis	Sage oil (providing ALA)	Oils: 5% by weight of diet	21 days	No effect on DSS or TNBS colitis-induced histological changes Increased mucosal inflammation (DSS colitis only)
			Fish oil			Decreased colon length (DSS colitis only)No effect on TNBS colitis-induced histological changes
Zhao et al. [46]	Mice	IL-10 deficiency	DHA	35.5 mg/kg body weight per day intragastrically	14 days	Improved histological inflammation score
Chien et al. [47]	Male Wistar rats	Chronic ethanol exposure	Fish oil	7.1 or 16.2 g/kg diet	8 weeks	No effect on epithelial histological damage
Yao et al. [48]	Male Sprague–Dawley rats	TNBS colitis	ω-3 PUFAs (source not specified but presumed to be fish oil)	20 mg/kg body weight per day intragastrically	60 days	Decreased disease activity index score Decreased colonic macroscopic damage index score (decreased ulceration) Decreased tissue damage index score (reduced thickening and leukocyte infiltration)
Charpentier et al. [49]	Young male Sprague–Dawley rats	TNBS colitis	ω-3 PUFAs (source not specified but presumed to be fish oil)	6.1 g/kg of diet	28 days	No effect on colonic weight to length ratio
Haddi et al. [50]	Female BALB/c mice	β-lactoglobulin-induced inflammation	Fish oil	0.6, 1 or 1.5 mL/kg body weight per day by gavage	15 days	Increased villus height Improved intestinal architecture Improved histological score
Tang et al. [51]	Female Sprague–Dawley rats	Peritoneal dialysis	ω-3 PUFAs (source not specified)	0.5 or 1.5 g/kg body wt per day intragastrically	28 days	Increased ileal villus length Increased crypt depth/ileal villus length ratio
Zheng et al. [52]	Male C57 mice	DSS colitis	DPA	300 mg/kg body weight per day by gavage	28 days	Attenuated body weight decrease Decreased disease activity index score Improved gross morphology and pathological inflammatory score Attenuated inflammatory infiltrationAttenuated colon shortening

Abbreviations used: ALA, α-linolenic acid; DHA, docosahexaenoic acid; DPA, docosapentaenoic acid; DSS, dextran sodium sulphate; EPA, eicosapentaenoic acid; LT, leukotriene; PUFA, polyunsaturated fatty acid; TNBS, 2,4,6-trinitrobenzenesulfonic acid.

**Table 4:** Effects of ω-3 PUFAs on intestinal permeability in cell and rodent models.

Reference	Model Used	Condition	ω-3 PUFA(s) Used	Concentration or Dose Used	Duration	Changes to Permeability and Related Mechanisms
Cell line models						
Rosella et al. [23]	Caco-2 cells	Non-stimulated	EPA	100 μg/mL	24 h	↓ Permeability
Usami et al. [59]	Caco-2 cells	Non-stimulated	ALA	50–200 μM	24 h	↑ Permeability (dose dependent)
			EPA			↑ Permeability (dose dependent) ↓ Electron-dense material at tight junctions and desmosomes (200 μM only)
Usami et al. [60]	Caco-2 cells	Non-stimulated	DHA	10–100 μM	24 h	↑ Permeability (dose dependent)
Willemsen et al. [26]	T84 cells	IL-4-induced inflammation	ALA	10 or 100 μM	48 h	No effect on permeability
			EPA			↓ Permeability (100 μM only)
			DHA			↓ Permeability (100 μM only)
Li et al. [27]	T84 cells	TNF-α- and IFN-γ-induced inflammation	EPA	25–75 μM	48 h	↓ Permeability ↓ Tight junction protein redistribution ↓ Tight junction altered morphology ↓ Occludin and flotillin displacement from lipid rafts
			DHA			↓ Permeability ↓ Tight junction protein redistribution ↓ Tight junction altered morphology ↓ Occludin and flotillin displacement from lipid rafts
Xiao et al. [24]	Caco-2 cells	Heat stress	EPA	50 μM	96 h	↓ Permeability ↓ Tight junction altered morphology ↓ Tight junction protein redistribution ↑ ZO-1 and occludin protein and mRNA expression
			DHA			No effect on permeability ↑ ZO-1 and occludin protein and mRNA expression
Xiao et al. [61]	IPEC-1 cells	Deoxynivalenol-induced inflammation	EPA	Up to 25 μg/mL	24–72 h	↓ Permeability (24 and 48 h) ↓ ZO-1 and claudin redistribution
			DHA			↓ Permeability (24 and 48 h) ↓ ZO-1 and claudin redistribution
Rodent models						
Empey et al. [38]	Male Sprague–Dawley rats	Acetic acid-induced colitis	EPA-enriched fish oil	10% by weight of total diet	6 weeks	Protected ileal and colonic absorption
Caplan et al. [41]	Neonatal Sprague–Dawley rats	Necrotising enterocolitis	DHA	23 mg/100 mL formula	96 h	↓ Plasma endotoxin level
Hudert et al. [36]	Transgenic fat-1 mice	DSS colitis	-	Mice have higher colonic EPA, DPA and DHA	-	↑ ZO-1 expression (maintained compared to colitis control)
Xiao et al. [33]	Male Wistar rats	Heatstroke	EPA	1 g/kg body weight/day by gavage	21 days	↓ Intestinal permeability ↓ Plasma endotoxin and D-lactate levels ↓ Tight junction protein distortion ↑ Tight junction protein expression
			DHA			↓ Intestinal permeability ↓ Plasma endotoxin and D-lactate levels ↓ Tight junction protein distortion ↑ Tight junction protein expression
Charpentier et al. [49]	Young male Sprague–Dawley rats	TNBS colitis	ω-3 PUFAs (source not specified but presumed to be fish oil)	6.1 g/kg of diet	28 days	No effect on claudin-1 protein expression No effect on occludin protein expression No effect on TTF3 protein expression No effect on MUC2 protein expression
Chien et al. [47]	Male Wistar rats	Chronic ethanol exposure	Fish oil	7.1 or 16.2 g/kg diet	8 weeks	↓ Plasma endotoxin levels ↑ ZO-1 immunoreactive area in intestinal epithelial tissue (16.2 g/kg/day only)

Abbreviations used: ALA, α-linolenic acid; DHA, docosahexaenoic acid; DPA, docosapentaenoic acid; DSS, dextran sodium sulphate; EPA, eicosapentaenoic acid; ETE, eicosatrienoic acid; IFN, interferon; IL, interleukin; PUFA, polyunsaturated fatty acid; TNBS, 2,4,6-trinitrobenzenesulfonic acid; TNF, tumour necrosis factor; ZO, zonula occludens.

**Table 5:** Effects of ω-3 PUFAs on inflammatory mediators in cell, rodent and human models.

Reference	Model Used	Condition	ω-3 PUFA(s) Used	Dose Used	Duration	Effect on Inflammatory Mediator(s)
Cell line models						
Zhao et al. [66]	HCT116 cells	Lauric acid/IE-DAP/MDP-induced inflammation	EPA	0–20 μM	20 h	↓ IL-8 protein (MDP only)
			DHA			↓ IL-8 protein (all treatments)
Marion-Letellier et al. [64]	Caco-2 cells	IL-1β-induced inflammation	EPA	0.1–10 μM	18 h	↓ IL-6 protein ↓ IL-8 protein
			DHA			↓ IL-6 protein ↓ IL-8 protein
Vincentini et al. [67]	Caco-2 cells	α-gliadin-induced inflammation	DHA	2 μM	24 h	↓ PGE_2_ ↓ IL-8 protein
Bentley-Hewitt et al. [63]	HT29/HT29-MTX cell co-culture	Non-stimulated	EPA	50 μM	12 h	↑ TGF-β1 mRNA No consistent effect on IL-8 or HSP 72 mRNA
			DHA			↑ TGF-β1 mRNA No consistent effect on IL-8 or HSP 72 mRNA
Reifen et al. [32]	Caco-2 cells	IL-1β-induced inflammation	Sage oil (providing ALA)	10 μM	48 h	↓ IL-8 protein
			ALA			↓ IL-8 protein
Wijendran et al. [65]	H4/NEC-IEC/Caco-2 cells	IL-1β-induced inflammation	EPA	100 μM	48 h	↓ IL-8 mRNA and protein (H4 only) ↓ IL-6 mRNA and protein (H4 only)
			DHA			↓ IL-8 mRNA and protein ↓ IL-6 mRNA (H4 only) ↓ IL-6 protein (H4 and NEC-IEC)
Rodent models						
Empey et al. [38]	Rats	Non-stimulated	EPA-enriched fish oil	10% by weight of total diet	6 weeks	↑ PGE_2_ in colonic dialysate ↑ LTB_4_ in colonic dialysate
Yuceyar et al. [40]	Male Wistar albino rats	TNBS colitis	Fish oil	EPA: 14.4 mg/g diet DHA: 11.6 mg/g diet	6 weeks (diet)	↓ Colonic LTB_4_ ↓ Colonic LTC_4_
					14 days (daily enema)	↓ Colonic LTB_4_ ↓ Colonic LTC_4_
Andoh et al. [42]	Male Sprague–Dawley rats	TNBS colitis	ω-3 PUFA-rich liquid diet (providing ALA)	150 mg/100 kcal	12 days (followed by 2 days starvation)	↓ Mucosal IL-6 secretion No effect on mucosal TNF-α secretion
Hudert et al. [36]	Transgenic fat-1 mice	DSS colitis	-	Mice have higher colonic EPA, DPA and DHA than controls	-	↑ Mucosal RvE1 ↑ Mucosal RvD3 ↑ Mucosal protectin D1 ↑ Mucosal PGE_3_ ↑ Mucosal LTB_5_ No effect on mucosal LTB_4_ No effect on mucosal PGE_2_ No effect on mucosal 15-hydroxyeicosatetraenoic acid (lipoxin A_4_ precursor) ↓ Colonic TNF-α mRNA ↓ Colonic IL-1β mRNA ↑ Colonic toll-interacting protein mRNA ↑ Colonic trefoil factor 3 mRNA
Wang et al. [62]	Male Lewis rats	Non-stimulated	Fish oil	Fish oil 4% by weight of total diet (EPA: 15.4% of total fatty acids; DHA: 15.1% of total fatty acids)	up to 90 days	↓ TNF-α mRNA ↓ IFN-γ mRNA ↓ IL-4 mRNA ↓ IL-10 mRNA ↓ IL-15 mRNA and protein No effect on IL-7 mRNA or protein
Hassan et al. [44]	Male Sprague–Dawley rats	TNBS colitis	ALA	28.8% of total fat content of formula	2 weeks	↓ TNF-α mRNA and protein ↓ LTB_4_ No effect on IL-6 expression or secretion No effect on PGE_2_
Bosco et al. [30]	Rag2^−/−^ immunodeficient mice	Adoptive transfer of naïve T-cell-induced colitis	Fish oil	EPA: 3.37 g/100 g diet DHA: 2.10 g/100 g diet	8 weeks	↑ Colonic myeloperoxidase ↑ Colonic IL-1β protein ↑ Colonic IL-12 protein ↑ Colonic keratinocyte-derived chemokine protein ↑ Colonic IL-10 protein ↑ Colonic TNF-α protein ↑ Mucosal PGE_3_ ↑ Mucosal TXB_3_ ↑ Mucosal LTB_5_ ↑ Mucosal 5-HEPE ↑ Mucosal 17,18-EEP ↓ Mucosal PGJ_2_ ↓ Mucosal 5,6-EET ↓ Mucosal 8,9-EET ↓ Mucosal 14,15-EET No effect on mucosal PGE_2_ No effect on mucosal TXB_2_ No effect on mucosal LTB_4_
Brahmbhatt et al. [31]	Male Sprague–Dawley rats	Intestinal reperfusion and ischaemia	EPA and DHA	EPA: 3.00% of dietary fatty acids DHA: 1.98% of dietary fatty acids	3 weeks	No effect on cytokine production ↑ TXB_3_ ↑ 17,18-EEP ↑ 8-iso PGF_3α_
Zhao et al. [46]	Mice	IL-10 deficient	DHA	35.5 mg/kg body weight per day intragastrically	2 weeks	↓ TNF-α protein ↓ IFN-γ protein ↓ IL-17 protein
Charpentier et al. [49]	Young male Sprague–Dawley rats	TNBS colitis	ω-3 PUFAs (source not specified but presumed to be fish oil)	6.1 g/kg of diet	28 days	↓ Colonic IL-6 protein ↓ Colonic LTB_4_ No effect on colonic TNF-α protein
Yao et al. [48]	Male Sprague–Dawley rats	TNBS colitis	ω-3 PUFAs (source not specified but presumed to be fish oil)	20 mg/kg body weight per day intragastrically	60 days	↓ Colonic IL-2 mRNA ↓ Colonic IL-4 mRNA
Zheng et al. [52]	Male C57 mice	DSS colitis	DPA	300 mg/kg body weight per day by gavage	28 days	↓ Colonic IL-1β mRNA and protein ↓ Colonic IL-6 mRNA and protein ↓ Colonic TNF-α mRNA and protein ↑ Colonic IL-10 mRNA and protein ↓ Colonic PGE_2_ ↓ Colonic LTB_4_
Human studies						
Hillier et al. [34]	Human	Inflammatory bowel disease	Fish oil	Fish oil: 18 g/day (3.3 g EPA and 2.2 g DHA per day)	12 weeks	↓ Colonic mucosa PGE_2_ ↓ Colonic mucosa TXB_2_

Abbreviations used: ALA, α-linolenic acid; DHA, docosahexaenoic acid; DPA, docosapentaenoic acid; DSS, dextran sodium sulphate; EEP, epoxyeicosatetraenoic acid; EET, epoxyeicosatrienoic acid; EPA, eicosapentaenoic acid; HEPE, hydroxyeicosapentaenoic acid; HSP, heat shock protein; IE-DAP, γ-D-glutamyl-mesodiaminopimelic acid; IFN, interferon; IL, interleukin; LT, leukotriene; MDP, muramyldipeptide; PG, prostaglandin; PUFA, polyunsaturated fatty acid; Rv, resolvin; TGF, transforming growth factor; TNBS, 2,4,6-trinitrobenzenesulfonic acid; TNF, tumour necrosis factor; TX, thromboxane.

**Table 6:** Effects of ω-3 PUFAs on inflammatory mechanisms in rodent and cell models.

Reference	Model Used	Condition	ω-3 PUFA(s) Used	Concentration or Dose Used	Duration	Effect on Inflammatory Mechanisms(s)
Cell line models						
Hofmanová et al. [70]	HT-29 cells	TNF-α or anti-Fas monoclonal antibody/cycloheximide-induced inflammation	DHA	20 μM	48 h	↑ G_0_/G_1_ phase cells ↑ Apoptosis (TNF-α and anti-Fas monoclonal antibody treatments)
Renaville et al. [28]	T84/Caco-2 cells	Non-stimulated	EPA	300 M	3 h or 3 h and 7 days	No effect on PPAR-α mRNA (both time periods) ↓ Stearoyl CoA desaturase and SREBP-1c mRNA (3 h and 7 days only)
Lu et al. [43]	IEC-6 cells	Platelet-activating factor treatment	DHA	67 μM	30 min	↓ TLR4 mRNA ↓ Platelet-activating factor receptor mRNA
Zhao et al. [66]	HCT116 cells	Lauric acid/IE-DAP/MDP-induced inflammation	DHA	0–20 μM	20 h	↓ NF-κB activation ↓ IκB degradation
			EPA			No effect on NF-κB activation No effect on IκB degradation
Marion-Letellier et al. [64]	Caco-2 cells	IL-1β-induced inflammation	ALA	0.1–10 μM	18 h	No effect on PPAR-γ protein No effect on iNOS protein No effect on IκB protein
			DHA			↓ PPAR-γ protein ↓ iNOS protein No effect on IκB protein
			EPA			↓ PPAR-γ protein ↓ iNOS protein No effect on IκB protein
Vincentini et al. [67]	Caco-2 cells	α-gliadin-induced inflammation	DHA	2 μM	24 h	↓ Cytosolic phospholipase 2 activity ↓ COX-2 protein
Kimura et al. [68]	Caco-2 cells	Non-stimulated	DHA	25 μM	24 h	↑ PPAR-α activity ↑ PPAR-γ activity No effect on PPAR-δ activity ↓ Triglyceride and apolipoprotein B secretion
			EPA			↑ PPAR-α activity No effect on PPAR-γ activity No effect on PPAR-δ activity
Reifen et al. [32]	Caco-2 cells	IL-1β-induced inflammation	Sage oil	10 μM	48 h	↓ COX-2 protein
			ALA			↓ COX-2 protein ↓ iNOS protein
Wijendran et al. [65]	H4/NEC-IEC/Caco-2 cells	IL-1β-induced inflammation	DHA	100 μM	48 h	↓ NF-κB mRNA ↓ IL-1R1 mRNA
			EPA			No effect on NF-κB mRNA No effect on IL-1R1 mRNA
Rodent models						
Yuceyar et al. [40]	Male Wistar albino rats	TNBS colitis	Fish oil	EPA: 14.4 mg/g diet DHA: 11.6 mg/g diet	6 weeks (diet)	↓ Myeloperoxidase activity
					14 days (daily enema)	No effect on myeloperoxidase activity
Caplan et al. [41]	Neonatal Sprague–Dawley rats	Necrotising enterocolitis	DHA	23 mg/100 mL formula	96 h	No effect on iNOS protein ↓ Phospholipase A_2_ protein ↓ Platelet-activating factor receptor protein
Hudert et al. [36]	Transgenic fat-1 mice	DSS colitis	-	Mice have higher colonic EPA, DPA and DHA than controls	-	↓ Colonic NF-κB activity ↓ Colonic iNOS mRNA
de Vogel-van den Bosch et al. [71]	129S1/SvImJ wild-type mice	Non-stimulated	DHA	EPA or DHA: 12.5 g/kg body weight by gavage	6 h	No effect on regulated long-chain fatty acid uptake, mitochondrial and peroxisomal β-oxidation, ω-oxidation, and metabolism of energy-yielding substrates No effect on regulated oxidative stress mRNAs ↓ Cholesterol uptake transporter (Npc1l1), apical mannose and glucose uptake transporter (Sglt4), and serotonin transporter (Slc6a4)
			EPA			No effect on regulated long-chain fatty acid uptake, mitochondrial and peroxisomal β-oxidation, ω-oxidation, and metabolism of energy-yielding substrates No effect on regulated oxidative stress mRNAs ↓ Cholesterol uptake transporter (Npc1l1), apical mannose and glucose uptake transporter (Sglt4), and serotonin transporter (Slc6a4) ↑ Cholesterol efflux protein (Abca1) and dopamine transporter (Dat1)
Hassan et al. [44]	Male Sprague–Dawley rats	TNBS colitis	ALA	28.8% of total fat content of formula	2 weeks	↓ Colonic iNOS protein ↓ Colonic COX-2 protein ↓ Colonic NF-κB activation No effect on phosphorylation of JNK, P38 and IκB
Kimura et al. [68]	Male C57BL/6 mice	PPAR-α deficiency	DHA-rich oil	60% energy fat diet with1.9% or 3.7% of total fatty acids as DHA (plus some EPA)	1 week	↓ Triglyceride secretion (inhibited by PPAR-α deficiency) with 3.7% DHA
			EPA-rich oil	3.4% EPA and 1.5% DHA		No effect on triglyceride secretion
Reifen et al. [32]	Male Wistar rats	TNBS- or DSS-induced colitis	Fish oil	Fish oil: 5% by weight of total diet (EPA: 11.7% of total fatty acids DHA: 15.7% of total fatty acids)	3 weeks	↓ COX-2 mRNA
			Sage oil	Sage oil: 5% by weight of total diet (ALA: 43.9% of total fatty acids)		↓ COX-2 mRNA
Charpentier et al. [49]	Young male Sprague–Dawley rats	TNBS colitis	ω-3 PUFAs (source not specified but presumed to be fish oil)	6.1 g/kg of diet	28 days	↑ Colonic IL-1A mRNA ↑ Colonic TLR-2 mRNA ↑ Colonic mitogen-activated protein kinase kinase 3 mRNA ↓ Colonic iNOS protein ↓ Colonic COX-2 protein
Yao et al. [48]	Male Sprague–Dawley rats	TNBS colitis	ω-3 PUFAs (source not specified but presumed to be fish oil)	20 mg/kg body weight per day intra-gastrically	60 days	↓ Colonic nuclear factor of activated T cells mRNA ↑ Colonic PPAR-γ mRNA
Zheng et al. [52]	Male C57 mice	DSS colitis	DPA	300 mg/kg body weight per day by gavage	28 days	↓ Colonic myeloperoxidase activity ↓ Colonic COX protein ↓ Colonic 5-LOX protein

Abbreviations used: ALA, α-linolenic acid; COX, cyclooxygenase; DHA, docosahexaenoic acid; DPA, docosapentaenoic acid; DSS, dextran sodium sulphate; EPA, eicosapentaenoic acid; IE-DAP, γ-D-glutamyl-mesodiaminopimelic acid; IκB, inhibitory subunit of NF-κB; IL, interleukin; LOX, lipoxygenase; MDP, muramyldipeptide; NF-κB, nuclear factor kappa-light-chain-enhancer of activated B cells; NOS, nitric oxide synthase; PPAR, peroxisome proliferator activated receptor; PUFA, polyunsaturated fatty acid; SREBP, sterol receptor element-binding protein; TLR, toll-like receptor; TNBS, 2,4,6-trinitrobenzenesulfonic acid; TNF, tumour necrosis factor.

## Data Availability

Not applicable.
